# Abnormal myocardial T1 mapping of hypertrophic cardiomyopathy in areas without delayed enhancement, as compared to NICM and controls at both 1.5 and 3T

**DOI:** 10.1186/1532-429X-18-S1-Q45

**Published:** 2016-01-27

**Authors:** Orhan Sancaktar, Kaushik Shahir, Dhiraj Baruah, Aimee C Welsh, Jason Rubenstein

**Affiliations:** 1 Cardiovascular Medicine, Medical College of Wisconsin, Denver, CO USA; 2 Radiology, Medical College of Wisconsin, Milwaukee, WI USA

## Background

The use of T1 mapping for myocardial tissue characterization and calculation of extracellular volume using Modified Look-Locker inversion recovery (MOLLI) has been well described. Our goal was to apply the MOLLI technique to the septal myocardium of patients with known HCM and compare these to a control as well as a heterogeneous nonischemic cardiomyopathy (NICM) population.

## Methods

55 patients were included in the study; 29 with clinically proven hypertrophic cardiomyopathy, 10 patients with no cardiac disease, and 16 patients with nonischemic cardiomyopathy. All underwent cardiac MRI at either 1.5 or 3T (Verio or Espree, Siemens) including contrast using Magnevist (Bayer HealthCare) or Multihance (Bracco Imaging). T1 maps were obtained before and 10 minutes after contrast infusion for calculation of λ as previously described. Delayed steady-state free procession images were obtained. Post-processing was performed using CVI42 (Circle Imaging). For MOLLI calculation, T1 times were measured in the basal, mid, and apical regions of the septal myocardium avoiding areas with delayed hyperenhancement. Statistical analysis was performed using R Statistical Software (Foundation for Statistical Computing, Vienna, Austria).

## Results

There was no significant difference between the λ of basal, mid, and apical septal segments of HCM patients (0.45 vs 0.46 vs 0.47 p = 0.53). The septal λ was statistically different between HCM and controls at the base (0.45 vs 0.40 p = 0.005), mid (0.46 vs 0.41 p < 0.001), but not the apex (0.47 vs 0.43 p = 0.09). There was a statistically significant difference between the λ values of the apical myocardium when comparing the HCM and NICM groups as well (0.45 vs 0.51 p = 0.007).

## Conclusions

The septal myocardium of patients with hypertrophic cardiomyopathy displays abnormal λ implying an abnormal extracellular volume. Even without visible delayed enhancement, λ was able to differentiate HCM from both normal patients as well as patients with NICM. This may be an important diagnostic tool to identify early HCM patients prior to the development of overt delayed enhancement.

**Figure 1:**
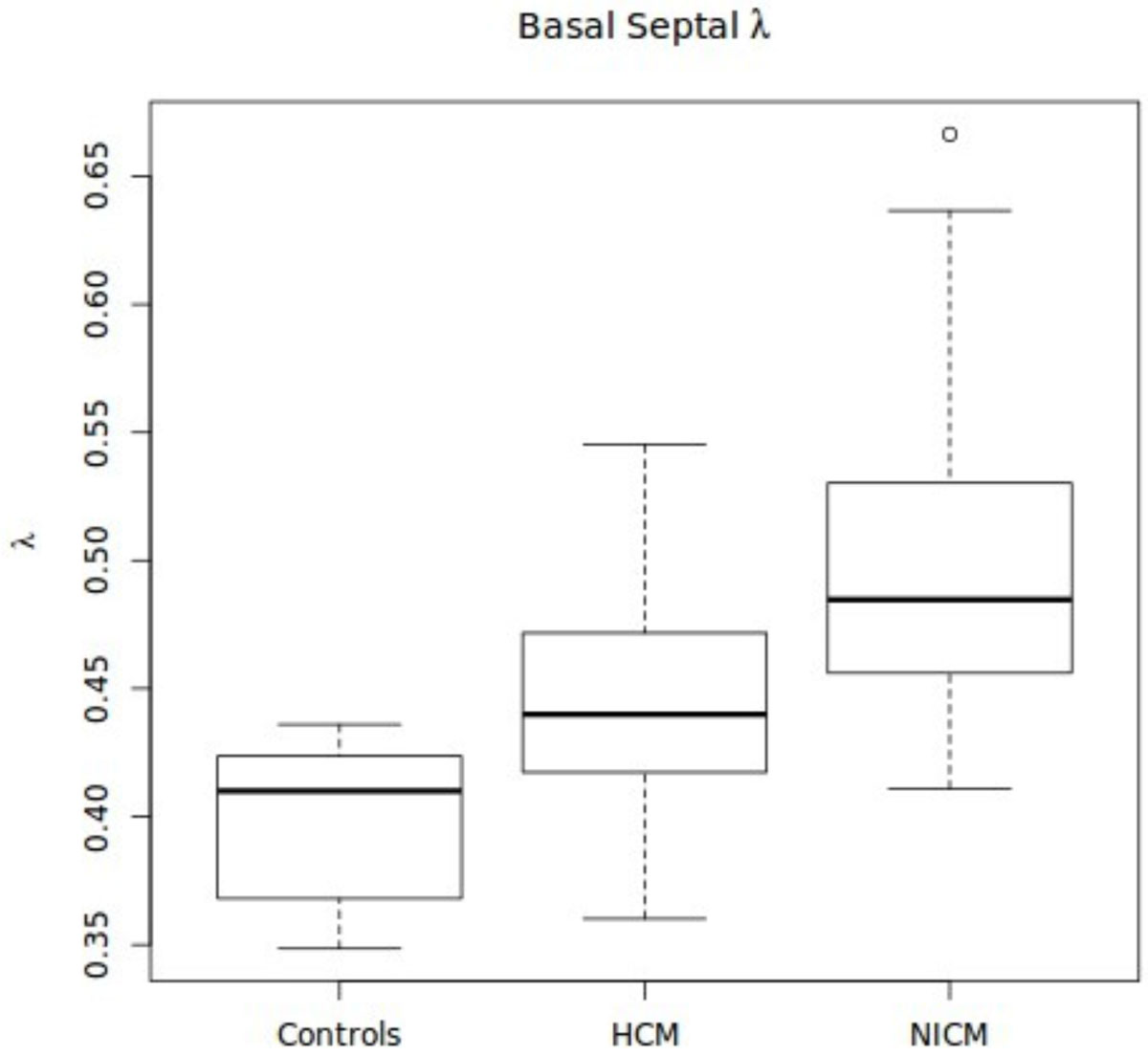
**Basal septal λ values for each of the control, HCM, and NICM populations**.

